# Volatile Composition and Sensory Characterization of Dry White Wines Made with Overripe Grapes by Means of Two Different Techniques

**DOI:** 10.3390/foods11040509

**Published:** 2022-02-10

**Authors:** Pau Sancho-Galán, Antonio Amores-Arrocha, Víctor Palacios, Ana Jiménez-Cantizano

**Affiliations:** Department of Chemical Engineering and Food Technology, Vegetal Production Area, Faculty of Sciences, Agrifood Campus of International Excellence (ceiA3), University of Cadiz, P.O. Box 40, 11510 Puerto Real, Spain; pau.sancho@uca.es (P.S.-G.); victor.palacios@uca.es (V.P.); ana.jimenezcantizano@uca.es (A.J.-C.)

**Keywords:** grape over-ripening, alcoholic fermentation, warm climate, climate change, wine aroma, volatile compounds, gas chromatography

## Abstract

Grape over-ripening is a technique that has historically been used for the production of white wines in southern Spain. However, this technique is still widely used for the production of sweet wines. In this study and after recently proving the feasibility of making dry white wines from overripe grapes with and without the presence of grape skin in a warm climate zone, the sensory characterization and analysis of the major and minor volatile compounds in dry white wines made from overripe grapes are presented for the first time. Two over-ripening techniques (sun-drying and climatic chamber drying) were studied for two different periods of time (48 and 96 h), as has the presence of grape skins during alcoholic fermentation. Grape over-ripening implies modifications in the composition of both the major and minor volatile compounds in wines. In terms of sensory analysis, wines with a similar profile were obtained year-on-year. The results of the preference test show that the wines made from grapes that had been over-ripened in the sun for 96 h were preferred by the tasting panel for both vintages. Thus, grape over-ripening under the sun could be considered as a resilience and adaptation technique for increased temperature conditions during the ripening season caused by the effects of climate change.

## 1. Introduction

Viticulture is a key socioeconomic and cultural sector in many countries and regions worldwide, with a high economic impact in the network of all relevant industry branches of the supply and distribution chains [1]. Geographically, grapevines are historically cultivated on six out of seven continents, between latitudes 4 and 51 in the northern hemisphere and between latitudes 6 and 45 in the southern hemisphere and across a large diversity of climates (oceanic, temperate, continental, Mediterranean, etc.), with the majority occurring in temperate climate regions [2]. However, climate change is exerting an increasingly profound influence on vine phenology and grape composition, and ultimately affects winemaking, wine microbiology, and chemistry and sensory aspects [3]. Observed changes in 27 premium viticultural regions across the globe have shown an increase in the average growing season temperature of 1.3 °C from 1950 to 2000, while in Europe, an increase of 1.7 °C was observed from 1950 to 2004 [4,5,6]. According to HadCM3 model average, the predicted temperatures for high-quality wine producing regions will increase by 2.04 °C within the period from 2000 to 2049 [5]. Understanding the changing suitability of regions for viticulture under climate change will help to us develop adaptation strategies in traditional winegrowing regions [7]. In order to maintain profitability and to ensure long-term future, producers will be required to adapt to changing climatic characteristics. Some of the guidelines for feasible adaptation strategies in the short term have been taken up in the Clim4Vitis action and include, among others, crop cultural measures [8,9,10], protection against extreme heat and sunburn [11,12,13], irrigation [14,15], pest and disease control [16,17], and soil management [18,19]. On the other hand, there are also long-term adaptation strategies such as changes in training systems [20,21], varietal/clonal and rootstock selection [22,23], or vineyard relocation [24,25,26]. The adoption of timely, cost-effective, and suitable adaptation strategies may significantly contribute to risk reduction, thereby decreasing the susceptibility of the sector and enhancing its resilience under a changing climate [27].

Given the foregoing precedents, quality wine production could be affected in those areas that already have a warm climate [28]. In this sense, another strategy to adapt to climate change-associated effects could be the application of traditional methodologies for the production of new types of wines other than traditional ones, taking advantage of the new conditions that have been imposed due to climate change, e.g., the production of dry white wines from overripe grapes [28]. Grape over-ripening is a method used in the production of raisins in countries such as India and China [29], but, in turn, it is also used in the hottest and driest countries of the world for the production of certain sweet and fortified wines [28]. In Andalusia (Southern Spain), special sweet wines are obtained using grapes that have been dried by direct exposure to the sun. While grape sugar enrichment can be achieved through the over-ripening of grapes on vines by twisting their stems without cutting them off, the traditional system used in Andalusia (Southern Spain) is the so-called asoleo technique, which consists of drying grape bunches in sun for several days in order to partially dry or raisin the grapes [30]. When subjected to hours of intense sunshine, grapes gradually lose water, resulting in a significant increase in the sugar concentration and a variation in the aromatic profile of the grapes [31]. However, this traditional system is susceptible to climatological variations that can alter the final product, in particular, rains during this period can cause the grapes to rot. It is useful to devise an alternative s over-ripening system that allows for greater control of the process [32] but that has no negative influence on the sensorial properties of final product. In recent years, a possible alternative to the traditional sun drying technique has appeared. Forced convection with hot air inside drying chambers is being used for the drying of horticultural products [33]. Climatic chambers for grape raisining [34,35] allow for the temperature and humidity to be controlled y, reduces the length of the required drying time, and makes the process independent of external meteorological conditions [36]. Nevertheless, grape over-ripening, regardless of the technique used, allows for natural modifications in the grape composition and leads to the production of new types of wines [28]. 

Present tendencies in wine consumption focus on well-structured wines that are full-bodied in the mouth [36]. Wine flavour is a combined perception of taste and aroma, the latter being the most responsible for the global perception of wines [33,37]. Wine aroma compounds can be grouped according to their origin: varietal aromas found in grapes, fermentative aromas from alcoholic and malolactic fermentation, and aging aromas obtained during aging or storage [38]. Their presence or absence in a particular wine depends on several factors, such as the environment (climate and soil), ripeness and grape variety, winemaking conditions, and wine aging [39,40,41]. Many of the volatile compounds that are generated during alcoholic fermentation are produced via the metabolic activity of *Saccharomyces cerevisiae* and quantitatively account for the biggest fraction of the total aroma composition of wine [38,42,43,44]. The extent to which these compounds persist from the grapes through to the finished wine is influenced by the winemaking conditions and the aging process [45]. The volatile fraction of wine is determined by several hundreds of chemically different compounds. Alcohols, aldehydes, esters, acids, monoterpenes, and other minor compounds usually constitute the volatile fraction of this product. 

New wine consumers are demanding more particular and exclusive wines that stand out from the rest because of their distinctive organoleptic characteristics [45]. Diversifying and innovating white wine production in a warm climate region by recovering historical winemaking techniques such as grape over-ripening and Grape Skin (GS) fermentation can be a way to bring together the search for new winemaking procedures as strategies to cope with the effects associated with climate change [28]. Thus, in this study and after recently proving the feasibility of making dry white wines from overripe grapes with and without the presence of GS in a warm climate zone [28], the sensory characterization and analysis of the major and minor volatile compounds in dry white wines made from overripe grapes using two techniques, sun and climatic chamber over-ripening, are presented for the first time.

## 2. Materials and Methods

‘Palomino Fino’ grapes, an autochthonous cultivar from a warm climate region [46], were harvested from a privately owned vine plot located at 36°64′29.7′′ N, 5°49′53.5′′ W at 150 m ASL in San José del Valle (Cadiz, Spain). Neither fertilisation nor irrigation treatments were applied in the vine plot during the two years of study. The experimental layout that was followed was similar to those that have been recently published [28,47]. Two different over-ripening techniques were studied: on one side, Sun-Drying (SD), and on the other side, Climatic Chamber drying (CH) at 35 ± 1 °C and with 10% relative humidity, in an Ibercex ASL climatic chamber (Madrid, Spain) located in the Institute of Viticulture and Agri-food Research (IVAGRO) of Cadiz University, in order to compare the over-ripening behaviour under controlled conditions. In both cases, two times were studied, 48 and 96 h, resulting in four different samples and a control without over-ripening in duplicate for each vintage studied (2018 and 2019). Grape ripeness after the grape over-ripening procedures was expressed as °Bé: Control: 11.300 ± 0.140, SD48h: 12.800 ± 0.140, SD96h: 13.500 ± 0.140, CH48h: 12.800 ± 0.000, and CH96h: 15.000 ± 0.140 for the 2018 vintage. Regarding the 2019 vintage, the °Bé values were as follows: Control: 12.180 ± 0.020, SD48h: 12.770 ± 0.040, SD96h: 13.910 ± 0.090, CH48h: 14.210 ± 0.060, and CH96h: 15.680 ± 0.030. Additionally, the grape composition required for this experiment and can be found in Sancho-Galán et al., 2021 [28]. In this sense, for each vintage, the experiment included 10 different fermentations (control without over-ripening, SD, and CH 48 and 96 h each, in duplicate) without GS and the same exact layout with the presence of 20% GS in order to study their effect on white winemaking. The different grape musts obtained were acidified with tartaric acid, and 80 mg/L of potassium metabisulphite was added as an antioxidant (Agrovin, Ciudad Real, Spain). For grape must fermentation, Lalvin 71B^®^ (Lallemand, Barcelona, Spain) was employed as a pre-ferment. Alcoholic Fermentation (AF) was carried out under controlled conditions at 18 °C in 5 L glass-made tanks, and as soon as it was completed, the wines were fined with 4 g/hL of gelatin and 40 g/hL of bentonite. After 72 h, the final wines were filtered, bottled, and corked. 

### 2.1. Analysis of Volatile Compounds

The methodology and equipment employed to determine the major volatile compounds were the same as those proposed by Amores-Arrocha et al. [48] and Sancho-Galán et al. [49]. Gas chromatography with flame ionisation detection (GC-FID, HP 5890 Series II) on a Carbowax 20 M column (L 50 m, ID 0.25 mm, PD 0.25 µm) was employed to determine major volatile compounds. The injector and detector temperatures were 175 °C and 225 °C, respectively, using hydrogen (1 mL/min) as a carrier gas. The oven temperature was 35 °C for the first 5 min, with a ramp of 5 °C/min until the temperature reached 100 °C. A direct injection of 5 µL of distilled sample was employed. Acetaldehyde, ethyl acetate, methanol, 2-propanol, and 2-methyl-1-propanol were determined using 4-methyl-2-pentanol (Sigma-Aldrich Química, S.A., Madrid, Spain) as an internal standard to determine retention times and calibration curves. 

Free minor volatile compounds were identified and quantified by semi-quantitative GC-MS analysis after the Solid Phase Extraction (SPE) of the different samples following the method described by Di Stefano [50]. The compound 1-Heptanol was used as an internal standard. The GC-MS methodology and specifications were the same as those reported in Amores-Arrocha et al. [48]. A GC-MS model Voyager^®^ (Termoquest, Milan, Italy) was used with a Supelcowax-10 column (L 60 m, ID 0.32 mm, PD 0.5 µm). The operation conditions were as follows: injector and detector temperature, 300 °C; oven temperature, 40 °C for 5 min followed by a 2 °C/min ramp and 200 °C for 5 min; sample volume, 2 µL in splitless mode (40 s); He as carrier gas at a 1 mL/min flow. The MS conditions were as follows: electronic impact mode (EI +) at 70 eV; initial temperature, 220 °C; interface temperature, 320 °C, scan index, 1 scan/s; mass acquisition range 45–400 *m/z.* Semi-quantitative analyses were carried out by assuming a response equal to one.

### 2.2. Sensory Analysis

A sensory analysis of the different wines produced during the two vintages was performed in order to determine the differences between the over-ripening technique, its time, and the presence or absence of GS. The wines were tasted 5 days after bottling by a 20-member panel comprising 12 women (30–54 years old) and 8 men (32–56 years old) who were experienced with wine tasting methodology. Informed consent was obtained from all the subjects involved in the study. An amount of 50 mL of wine was served to each taster in standard tasting glasses [51] at room temperature (22 ± 2 °C). Each of the glasses was randomly coded with a three-digit combination code and covered by a glass cover to prevent any of the volatile compounds from evaporating before the sensory analysis began. Additionally, the wines were presented to each panelist in a randomized order, and sample replicates were also assessed. Each panel member was provided with a specific tasting file comprising the olfactory and taste attributes selected according to Jackson [52], with scores to be evaluated on a 0- to 10-point scale, with 0 points representing the lowest score and 10 points representing the highest score. In accordance with the UNE-ISO-8587 standard [53], a preference test was carried out on the wines that were tasted in order to study the existence of significant differences in the different wines according to their elaboration methodology. To this end, the wines were grouped by vintage and by the presence or absence of GS in the fermentative medium, resulting in two preference tests of five wines each per vintage. The 20 tasters scored the wines from 1 to 5 according to their preference. The results of the preference analysis were calculated using Page’s preference test in accordance with the above-mentioned rule. 

### 2.3. Statistical Analysis

Means and standard deviations were calculated, and significant differences were evaluated by a two-way ANOVA and Bonferroni’s Multiple Range (BSD) test with a *p* < 0.05 using GraphPad Prism 6.01 (GraphPad Software, San Diego, CA, USA). The statistical analysis was performed on the volatile compounds obtained after wine analysis as well as on the results determined by the tasting panel.

## 3. Results and Discussion

**Figure 1:**
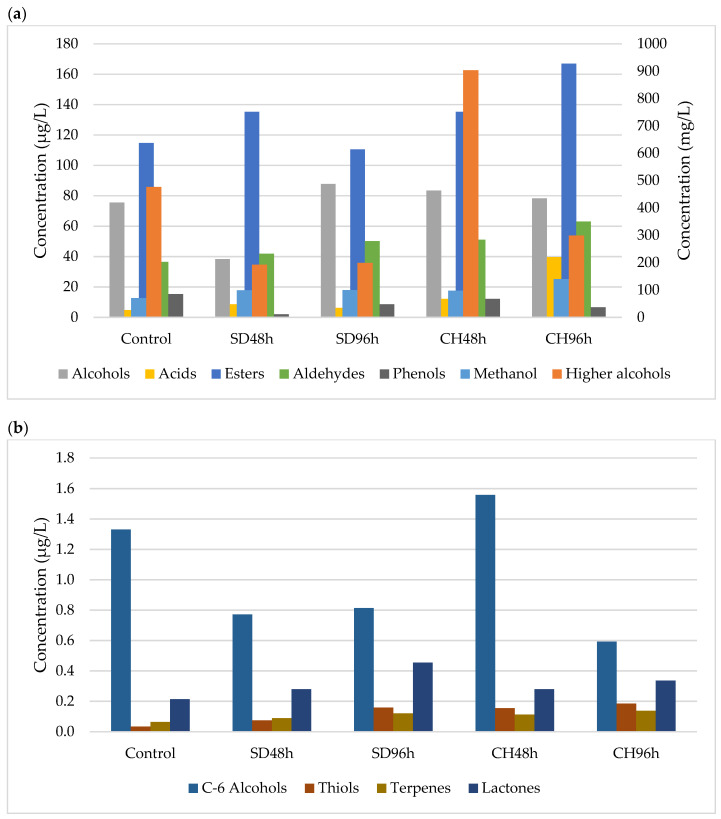
(**a**) Major and minor volatile compounds present in samples (*n* = 3) fermented without GS during 2018 vintage. Methanol and higher alcohols (mg/L, right axis). Alcohols, acids, esters, aldehydes and phenols, (µg/L, left axis). SD48h: sun-drying 48 h; SD96h: sun-drying 96 h; CH48h: climatic chamber drying 48 h; CH96h: climatic chamber drying 96 h. (**b**) Minor volatile compounds present in samples (*n* = 3) fermented without GS during 2018 vintage. SD48h: sun-drying 48 h; SD96h: sun-drying 96 h; CH48h: climatic chamber drying 48 h; CH96h: climatic chamber drying 96 h.

**Figure 2:**
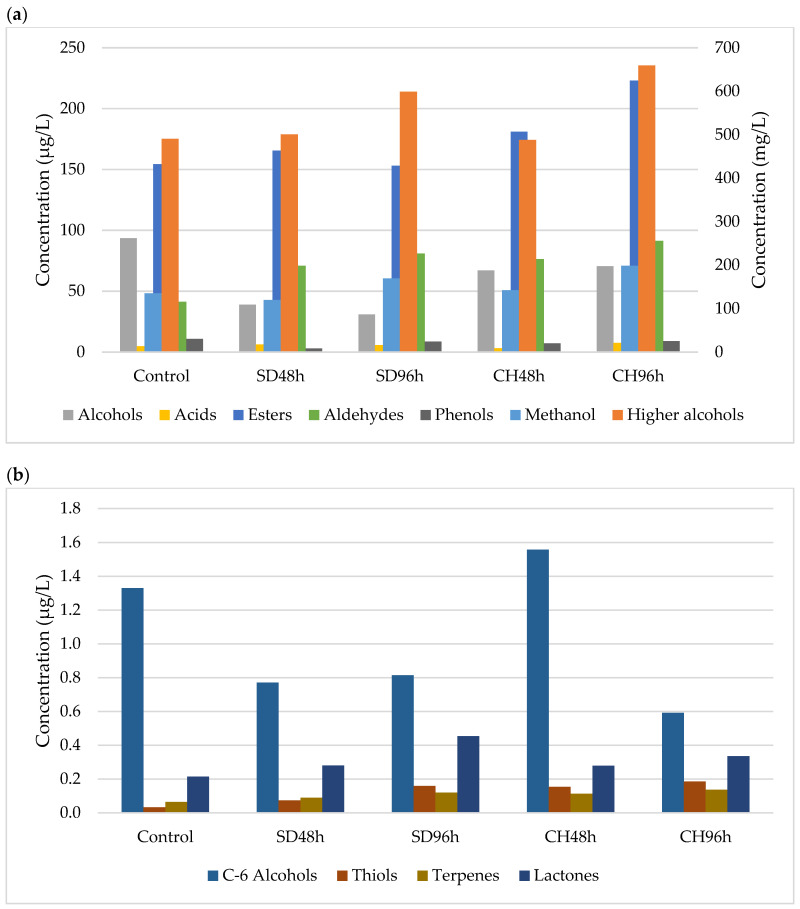
(**a**) Major and minor volatile compounds present in samples (*n* = 3) fermented with GS during 2018 vintage. Methanol and higher alcohols (mg/L, right axis). Alcohols, acids, esters, aldehydes and phenols, (µg/L, left axis). SD48h: sun-drying 48 h; SD96h: sun-drying 96 h; CH48h: climatic chamber drying 48 h; CH96h: climatic chamber drying 96 h. (**b**) Minor volatile compounds present in samples (*n* = 3) fermented with GS during 2018 vintage. SD48h: sun-drying 48 h; SD96h: sun-drying 96 h; CH48h: climatic chamber drying 48 h; CH96h: climatic chamber drying 96 h.

**Figure 3:**
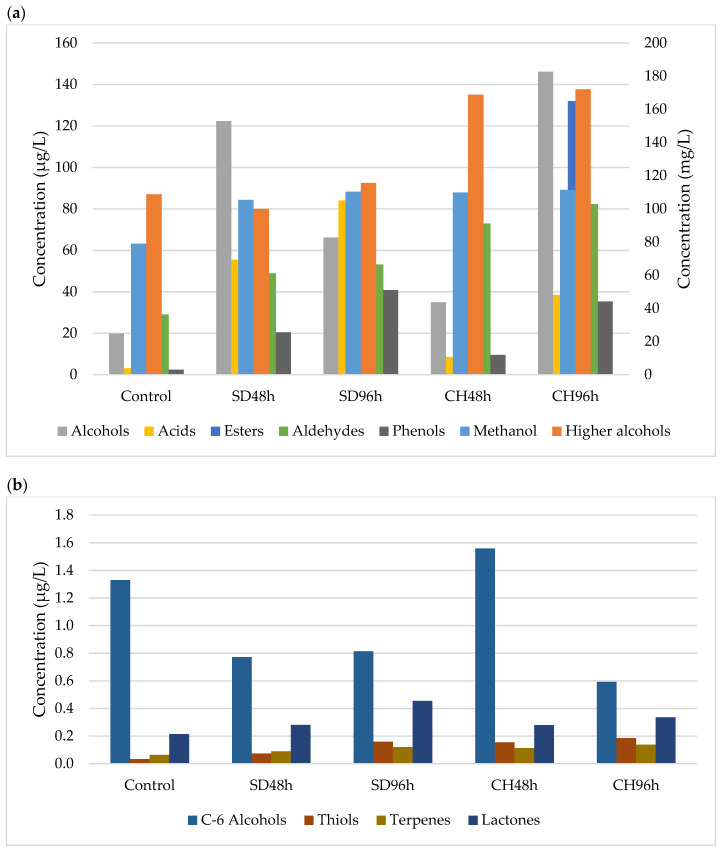
(**a**) Major and minor volatile compounds present in samples (*n* = 3) fermented without GS during 2019 vintage. Methanol and higher alcohols (mg/L, right axis). Alcohols, acids, esters, aldehydes and phenols, (µg/L, left axis). SD48h: sun-drying 48 h; SD96h: sun-drying 96 h; CH48h: climatic chamber drying 48 h; CH96h: climatic chamber drying 96 h. (**b**) Minor volatile compounds present in samples (*n* = 3) fermented without GS during 2019 vintage. SD48h: sun-drying 48 h; SD96h: sun-drying 96 h; CH48h: climatic chamber drying 48 h; CH96h: climatic chamber drying 96 h.

**Figure 4:**
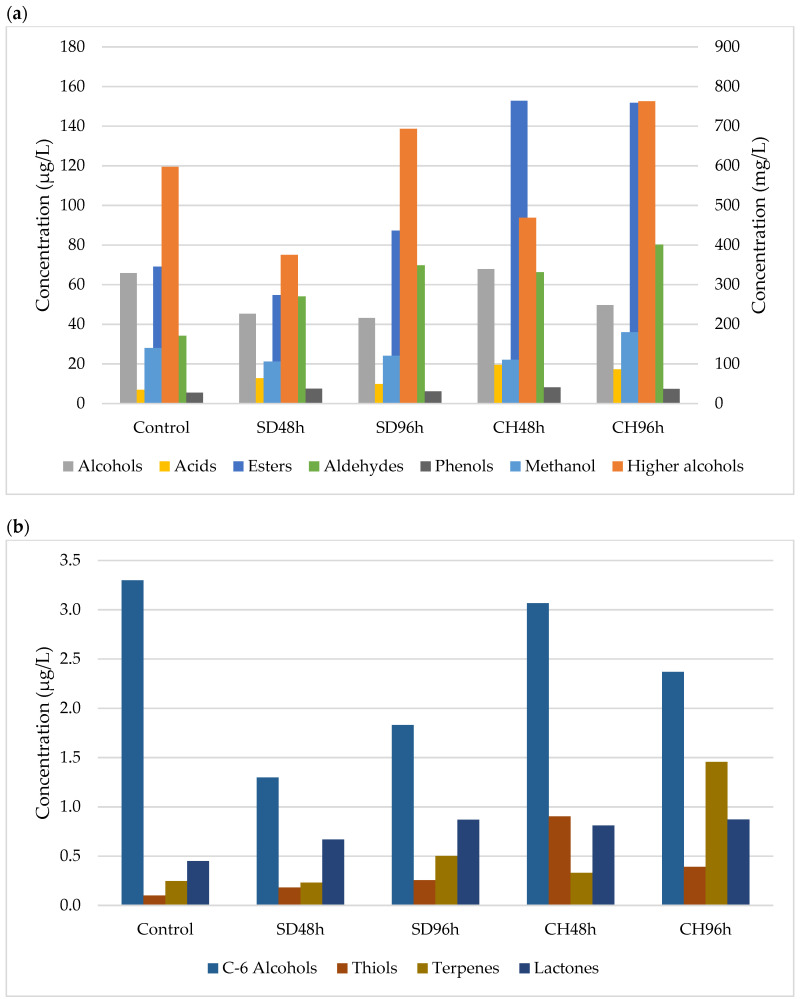
(**a**) Major and minor volatile compounds present in samples (*n* = 3) fermented with GS during 2019 vintage. Methanol and higher alcohols (mg/L, right axis). Alcohols, acids, esters, aldehydes and phenols, (µg/L, left axis). SD48h: sun-drying 48 h; SD96h: sun-drying 96 h; CH48h: climatic chamber drying 48 h; CH96h: climatic chamber drying 96 h. (**b**) Minor volatile compounds present in samples (*n* = 3) fermented with GS during 2019 vintage. SD48h: sun-drying 48 h; SD96h: sun-drying 96 h; CH48h: climatic chamber drying 48 h; CH96h: climatic chamber drying 96 h.

Figure 1, Figure 2, Figure 3 and Figure 4 show the effect of the applied over-ripening treatment and its duration and the presence or absence of GS on the profile of volatile compounds sorted by families during the two vintages studied (2018 and 2019). The statistical analysis and significant differences are reported as Supplementary Materials (Tables S1–S4).

### 3.1. Methanol and Major Alcohols

The methanol content observed in the two studied vintages and the different overripening and winemaking methodologies shows a predominant trend towards this analyte having higher concentrations in the wines made with the CH96h grapes (Figure 1, Figure 2, Figure 3 and Figure 4). After analysing the data, no correlation was observed between the different over-ripening treatments applied to the grapes, but there was a correlation with the hours of application: the methanol content was higher in the wines made with grapes that had been over-ripened for 96 h. However, it can be observed that the presence of grape skins in the fermentation medium produced an increase in the methanol concentration compared to wines produced without GS. This may be due to the contribution of the pectins from the skins, which are metabolized by the yeasts during the FAL, thus producing ethanol through enzymatic hydrolysis [48,49]. 

As for the major alcohols, this family of compounds represents the main part of the volatile compounds identified in the wines during the two vintages. Both 2-methyl-1-propanol and 2-propanol were identified in all of the wines produced, regardless of their methodology. In no case was there any correlation between the type of over-ripening treatment applied and the concentration of these secondary metabolites of the fermenting yeasts. Again, the presence of skins in the medium sponsored a higher concentration of major alcohols. This could be due to the Ehrlich catabolic pathway, where amino acids act as the precursors of volatile compounds [48]. This fact can be explained by the presence of skins, which implies an increase in the concentration of Free Amino Nitrogen (FAN) and therefore amino acids [28]. Despite that, the over-ripening procedures, which have also been shown to increase the amino acid concentration, did not lead to an increase in the major alcohol content in any of the cases studied. This second fact could be justified by the metabolic regulation of the yeast, where the absence of metabolic cofactors in the oxidised state such as NAD^+^ prevents the transformation of the intermediate aldehyde into a higher alcohol [54]. 

### 3.2. Volatile Alcohols and Acids

A total of 11 volatile minor alcohols were detected and showed fluctuations within the different samples and vintages, with no correlations being observed. This is mainly marked by fluctuations in the 2-phenylethanol and 2-nonanol contents. As for the volatile acid content, the same trend happened as with the alcohols: no clear trend was able to be observed between the different over-ripening treatments, application times, and vintages. However, for all of the cases studied, the concentration is significantly lower for the control wine (ANOVA *p* < 0.05; Tables S1–S4). This could be due to the fact that grape over-ripening implies an increase in their compounds due to water evaporation. This increase in fatty acids, among other compounds, can lead to an increase in the volatile acid content because an increase in the latter in grape musts can imply a lower degree yeast synthesis [55,56]. 

### 3.3. Esters

In all cases, the ester concentrations were found to be less than 1% of the total volatile compounds. It has been observed that regardless of the absence or presence of GS, wines with higher concentrations of these compounds were those that were made from grapes that had been over-ripened in a climatic chamber, specifically those that were subjected to this process for a longer period of time (96 h) (Figure 1, Figure 2, Figure 3 and Figure 4). Regarding the contribution of GS to the ester concentration, it was observed that their presence in the fermentation medium caused an increase in the concentration of these compounds. Ethyl acetate is the main compound that was observed, and the behaviour observed for the different samples during the two vintages was dependent on this compound to large extent.

Esters are compounds that are formed during the alcoholic fermentation of wines and play a fundamental role in wine aroma. They are of particular interest because they contribute to the series of fruity aromas [57]. The synthesis of these compounds, similar to volatile acids, is conditioned by the presence of fatty acids in the medium, which, together with alcohol, are the substrate for esterification reactions [48]. Thus, the higher the presence of fatty acids in the musts made from overripe grapes could explain the behaviour observed in the esters. 

### 3.4. Aldehydes

The aldehyde concentration increased significantly in all cases (ANOVA *p* < 0.05; Tables S1–S4) with respect to the control, and within each over-ripening treatment, it increased with the duration of the treatment as well as in the presence of GS (Figure 1, Figure 2, Figure 3 and Figure 4). The behaviour observed for this family of compounds is mainly due to acetaldehyde, which is the major compound in this group. However, in all cases, it is present in concentrations lower than 100 mg/L, so its contribution to the sensory profile of the wine is noticeable [48]. However, other compounds, such as benzeneacetaldehyde or valeraldehyde, can contribute to wines with nutty or floral notes due to their low perception thresholds [58].

### 3.5. C6-Alcohols

The compounds 1-Hexanol and (*Z*)-3-hexen1-ol appeared in a higher quantity in those wines that had been fermented in the presence of skins; however, no trend or correlation was observed in their concentration with respect to the over-ripening treatment or the time applied to the grapes in either of the two vintages studied. These compounds involve aromas of fresh herbs and vegetables [58,59], and their occurrence in greater quantities will depend on the presence of their precursors, linoleic and α-linoleic acids [60], in grape musts. Thus, the presence of skins and therefore the contribution of fatty acids [47] could explain the trend observed in the study samples over the two years. 

### 3.6. Phenols and Minor Compounds

The phenol content did not show any trend in relation to the over-ripening technique or its time and/or the presence of GS in the fermentation medium in any of the samples studied. These compounds, which may play an important role in the aromatic notes of the spice family, were found to have a high phenol content [61] that would have originated during AF via the decarboxylation of hydroxycinnamic acids by *Saccharomyces cerevisiae*.

As for the minor compounds, thiols (1-Propanol-3-metilthiol), terpenes (Linalool), and lactones (2,3-dihydro-benzofuranone) were detected. Regarding thiols, in all cases, the values were lower than 1 µg/L depending on the vintage and grape over-ripening time and methodology. An upward trend was observed for this compound with the hours of over-ripening, with the observed differences being significant in some cases (ANOVA *p* < 0.05; Tables S1–S4). Despite this increase, this compound of fermentative origin is related to the cysteine precursor content present in the musts that are degraded by yeasts [62]. The only terpene detected in the wine was Linalool, which showed higher concentrations in those wines fermented in the presence of grape skins, its concentration increasing with the hours that the grapes spent in the over-ripening process. This family of compounds gives floral notes to the wines [63], are of varietal origin, and are mainly found in the grape skins [64]; this would explain its higher presence in wines fermented in the presence of GS. Finally, the only lactone detected showed the same behaviour as terpenes. It could have originated during the wine alcoholic fermentation process and could have formed part of the aroma of the wine [65,66].

### 3.7. Sensory Analysis

**Figure 5:**
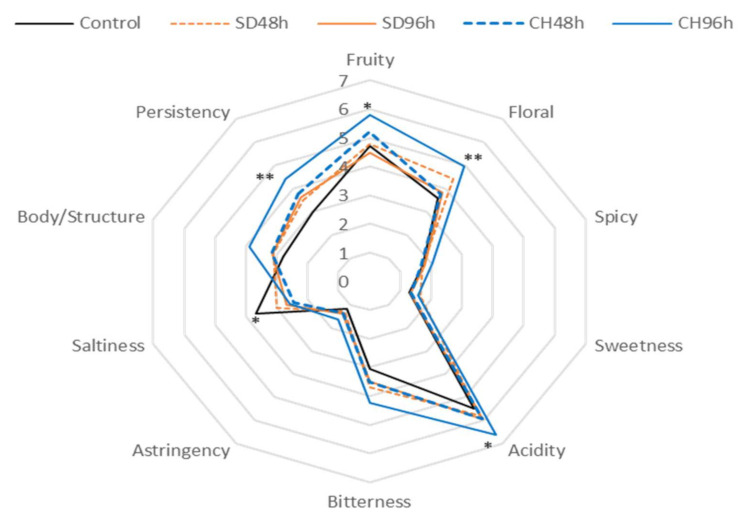
General attributes (aroma, taste, and mouth-feel properties) of the sensory analysis of wines fermented without GS during 2018 vintage. * Indicates level of significance for two-way ANOVA (BSD test) (* *p* < 0.05, ** *p* < 0.01). SD48h: sun-dried grapes during 48 h. SD96h: sun-dried grapes during 96 h. CH48h: climatic chamber drying during 48 h. CH96h: climatic chamber drying during 96 h.

**Figure 6:**
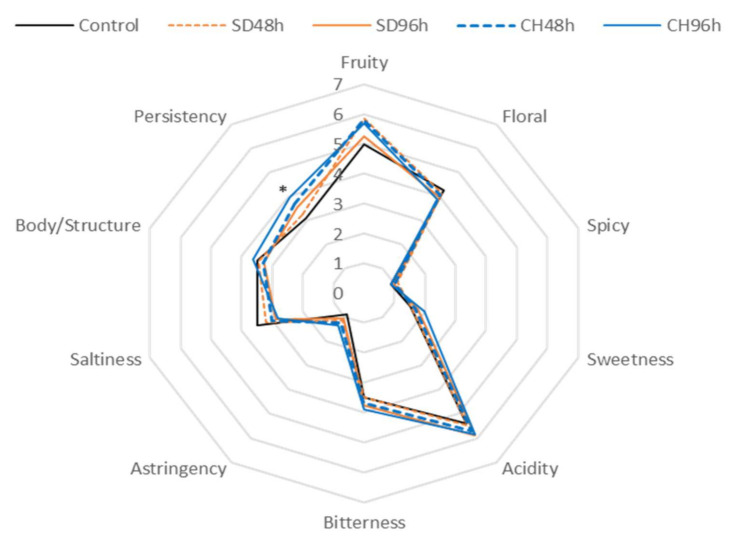
General attributes (aroma, taste, and mouth-feel properties) of the sensory analysis of wines fermented without GS during 2019 vintage. * Indicates level of significance for two-way ANOVA (BSD test) (* *p* < 0.05). SD48h: sun-dried grapes during 48 h. SD96h: sun-dried grapes during 96 h. CH48h: climatic chamber drying during 48 h. CH96h: climatic chamber drying during 96 h.

**Figure 7:**
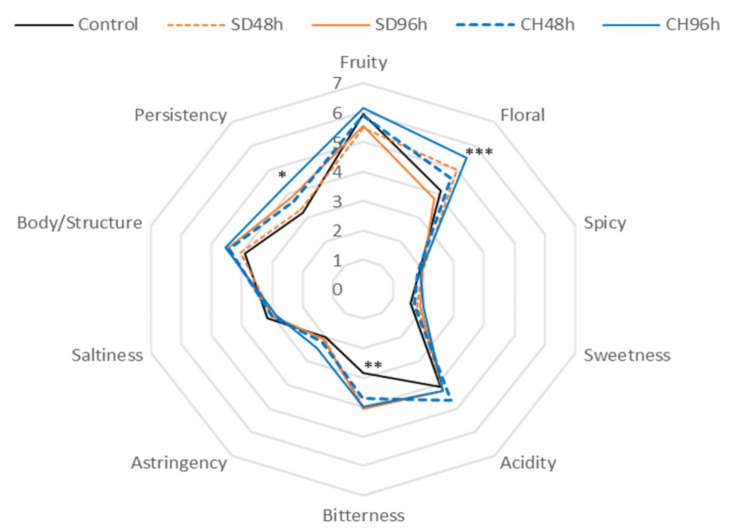
General attributes (aroma, taste, and mouth-feel properties) of the sensory analysis of wines fermented with GS during 2018 vintage. * Indicates level of significance for two-way ANOVA (BSD test) (* *p* < 0.05, ** *p* < 0.01, *** *p* < 0.001). SD48h: sun-dried grapes during 48 h. SD96h: sun-dried grapes during 96 h. CH48h: climatic chamber drying during 48 h. CH96h: climatic chamber drying during 96 h.

**Figure 8:**
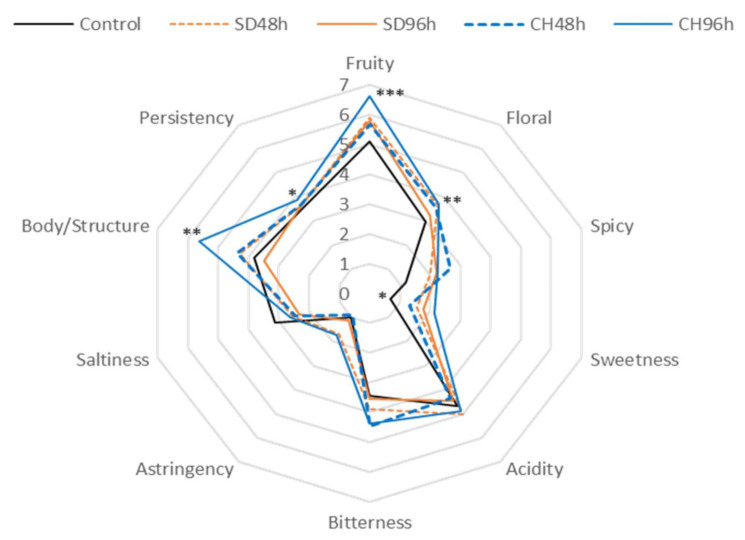
General attributes (aroma, taste, and mouth-feel properties) of the sensory analysis of wines fermented with GS during 2019 vintage. * Indicates level of significance for two-way ANOVA (BSD test) (* *p* < 0.05, ** *p* < 0.01, *** *p* < 0.001). SD48h: sun-dried grapes during 48 h. SD96h: sun-dried grapes during 96 h. CH48h: climatic chamber drying during 48 h. CH96h: climatic chamber drying during 96 h.

Figure 5, Figure 6, Figure 7 and Figure 8 show the results of the sensory analysis of all the wines made during the two vintages. 

In general terms, all of the wines showed an olfactory profile in which fruit and floral notes stand out, and in terms of taste, acidity and in some cases bitter notes are prominent. As for the wines made in the 2018 vintage, in both cases, a similar sensory profile is shown regardless GS were present in the fermentation tank. For wines made in absence of GS (Figure 5 and Figure 6), the CH96h wine showed significant differences (ANOVA *p* < 0.05) with respect to the control wine in the olfactory phase in terms of fruity and floral notes; the same differences in taste were observed in terms of persistence. The several parameters that were evaluated showed higher values in all cases in wines made with over-ripened grapes than in the control, probably due to the concentration effect exerted by the evaporation of the water from the grapes during grape over-ripening process [67]. This concentration can lead to an increase in those compounds that provide greater acidity and structure to the wine. In the same way, the wines made during 2019 without GS (Figure 6) behaved very similarly to those made in 2018, thus showing no effect of the vintage factor on the sensory profile of the wine. Once again, the different attributes that were evaluated showed higher scores in those wines made from overripe grapes, regardless of the over-ripening technique and time. However, in the latter case, significant differences were only observed in the persistence attribute with respect to the control.

As for the wines made with presence the presence of GS, similar behaviour was observed during the two vintages studied. Again, the wines showed a predominant floral aroma and acidity on the palate. With regard to the elaboration of GS presence in 2019, the differences found in the fruity and floral notes and in the body/structure and persistence of the CH96h wine with respect to the control wine stand out. It should also be noted, although not significantly, that this same wine presented higher sweetness values due to its final residual sugar content [28].

When comparing the presence and absence of skins within the same vintage, the presence of GS makes the wines more intense in terms of fruit and floral notes. This fact may be due to the increase in terpenes observed in Figure 5, Figure 6, Figure 7 and Figure 8 (and also in Tables S1–S4). The wines made with GS showed average acidity values, but lower than those produced conventionally, this fact was observed in previous research and may be due to the release of the Ca^2+^ and K^+^ cations by GS that help the precipitation of tartaric acid, resulting in a lower acid perception in wine sensory analysis [68]. The bitterness values were low in all of the analysed cases (3 out of 10 points); however, wines in the presence of GS had higher values, possibly due to the extraction of polyphenolic compounds, which can increase these perception values as well as in the body/structure and therefore the persistence of the wines [69,70,71,72].

Finally, the analysis of the preference test (UNE-ISO-8587) results showed significant differences in the tasters’ preferences for the two vintages according to the *F* values obtained through the Page test and are displayed on Table 1. 

For both vintages and with either the presence or absence of GS in the fermentation medium, the results of the preference test were significant in all cases, thus significantly indicating the preferences of the tasting panel. The best evaluated wine was the one made from grapes that had been over-ripened under the sun for 96 h (SD96h). According to the scores given by the tasting panel, those wines made with GS were significantly preferred to those made without GS, also during the two studied vintages. Thus, the CH96h wine, despite obtaining higher scores in terms of sensory perception of fruity and floral notes (Figure 5, Figure 6, Figure 7 and Figure 8), was not the preferred wine, probably because it presented higher sensations of bitterness in the tasting phase, thus devaluing its preference over the rest of the wines. 

## 4. Conclusions

In conclusion, grape over-ripening implies modifications regarding major and minor volatile compounds in wines. As expected, there are differences between grapes that have been over-ripened naturally or in the sun versus those that have been over-ripened in a climatic chamber under controlled conditions. In both cases, it was observed that the application time significantly affects the content of the volatile compounds, regardless of the ripening technique used. In addition, the presence of grape skins during alcoholic fermentation also produces differences in wines, resulting in wines with higher ester concentrations, which translates into wines with more floral and fruity notes.

In terms of sensory analysis, the wines were obtained with a very similar profile from one year to the other. In general terms, the wines made with overripe grapes had a significantly different sensory profile compared to the control wine. The wines that were obtained were dominated by fruity and floral notes. In addition, the results of the preference test showed that wines made from grapes that had been over-ripened under the sun for 96 h (SD96h) were preferred by the tasting panel during the two vintages studied. Based on the results obtained in this research work, it could be considered that the production of white wines from overripe grapes would help to diversify the production of quality white wines in a warm climate area. In turn, over-ripening under the sun could be considered as a resilience and adaptation technique for the increased temperature conditions during the ripening season caused by the effects of climate change.

## Figures and Tables

**Table 1:** *F* test values obtained after the preference test analysis for the two vintages.

	2018		2019	
	Without GS	With GS	Without GS	With GS
*F* test	354.6	392.6	385.6	397.0

## Data Availability

Supporting data are available at Sancho-Galán et al., *Foods* **2021**, *10*, 1583, https://www.mdpi.com/2304-8158/10/7/1583.

## Supplementary Materials

The following are available online at https://www.mdpi.com/article/10.3390/foods11040509/s1, Table S1: Volatile compound concentration (µg/L) in wines elaborated without GS during 2018 vintage, Table S2: Volatile compound concentration (µg/L) in wines elaborated with GS presence during 2018 vintage, Table S3: Volatile compound concentration (µg/L) in wines elaborated without GS during 2019 vintage, Table S4: Volatile compound concentration (µg/L) in wines elaborated with GS presence during 2019 vintage.
