# Supplementary material for: Volatile Composition and Sensory Characterization of Dry White Wines Made with Overripe Grapes by Means of Two Different Techniques
Source: Foods. 2022 Feb 10;11(4):509. doi: 10.3390/foods11040509 (PMC8871068; doi:10.3390/foods11040509)
Supplement: Supplementary file 1 [file foods-11-00509-s001.zip › foods-1591454-supplementary-done.pdf]

Table S1. Volatile compound concentration (μg/L) in wines elaborated without GS during 2018 vintage.

	Control	SD48h	SD96h	CH48h	CH96h
**Methanol**	7059.800 ± 10.249^{a}	9863.100 ± 12.961^{b}	9985.600 ± 12.389^{b}	9756.200 ± 5.976^{b}	13989.600 ± 12.679^{c}
% Methanol	12.53	16.40	14.22	16.58	17.43
**Higher alcohols**					
2-Methyl-1-Propanol	775.000 ± 0.106^{a,b}	123.300 ± 0.679^{a}	82.200 ± 0.016^{a,b}	65.200 ± 0.849^{a,b}	50.900 ± 0.596^{b}
2-Propanol	46852.000 ± 29.890^{a}	19120.500 ± 3.668^{b}	19834.100 ± 3.566^{b}	8968.700 ± 0.835^{b}	29864.700 ± 8.866^{a,b}
Total	47627.000 ± 29.996	19243.800 ± 4.347	19916.300 ± 3.583	9033.200 ± 1.684	29915.600 ± 9.462
% Higher alcohols	87.02	83.23	85.40	82.92	82.13
**Alcohols**					
3-Penten-2-ol	0.097 ± 0.005^{a}	0.750 ± 0.087^{b}	2.336 ± 0.319^{c}	0.074 ± 0.004^{a}	0.128 ± 0.013^{a}
1-Nonanol	0.178 ± 0.014^{a}	0.146 ± 0.011^{a}	0.155 ± 0.010^{a}	0.303 ± 0.008^{b}	0.190 ± 0.018^{a}
Benzyl alcohol	0.262 ± 0.020^{a}	0.124 ± 0.004^{b}	0.273 ± 0.009^{a}	0.484 ± 0.013^{c}	0.292 ± 0.013^{a}
2-Phenylethanol	71.272 ± 2.618^{a}	36.804 ± 0.067^{b}	76.758 ± 2.461^{a}	72.502 ± 1.078^{a}	70.321 ± 2.013^{a}
3-Ethoxy-1-Propanol	0.036 ± 0.001^{a,b}	0.050 ± 0.002^{a}	0.026 ± 0.004^{b,c}	0.016 ± 0.002^{c}	0.130 ± 0.011^{d}
2-Nonanol	1.439 ± 0.082^{a}	0.000 ± 0.000^{b}	6.167 ± 4.796^{c}	7.667 ± 0.697^{d}	6.040 ± 1.894^{c}
DL-2,3-Butanediol	1.143 ± 0.150^{a}	0.013 ± 0.001^{b}	1.102 ± 0.156^{a}	0.821 ± 0.116^{c}	0.022 ± 0.001^{b}
3-Ethyl-2-Pentanol	0.599 ± 0.055^{a,d}	0.177 ± 0.007^{b}	0.555 ± 0.002^{a}	0.780 ± 0.006^{c}	0.681 ± 0.040^{d,c}
3-Methyl-1-Pentanol	0.245 ± 0.021^{a,d}	0.029 ± 0.000^{b}	0.124 ± 0.009^{c}	0.287 ± 0.007^{a}	0.219 ± 0.012^{d}
4-Methyl-1-Pentanol	0.219 ± 0.022^{a,c}	0.219 ± 0.025^{a,c}	0.186 ± 0.005^{a}	0.288 ± 0.004^{b}	0.238 ± 0.011^{c}
1-Octanol	0.074 ± 0.007^{a}	0.049 ± 0.007^{b}	0.094 ± 0.003^{a}	0.155 ± 0.003^{c}	0.138 ± 0.003^{c}
Total	75.564 ± 2.997	38.349 ± 0.209	87.775 ± 7.774	83.376 ± 1.938	78.377 ± 4.028
% Alcohols	0.13	0.06	0.13	0.14	0.10
**Acids**					
Heptanoic acid	0.396 ± 0.028^{a}	0.128 ± 0.003^{b}	0.353 ± 0.002^{a}	0.368 ± 0.001^{a}	0.222 ± 0.020^{c}
2-Hexenoic acid	0.212 ± 0.017^{a}	0.064 ± 0.002^{b}	0.099 ± 0.007^{c}	0.200 ± 0.011^{a}	0.231 ± 0.027^{a}
3-Methylbutanoic acid	0.303 ± 0.032^{a}	0.046 ± 0.007^{b}	0.237 ± 0.020^{c}	0.165 ± 0.003^{d}	0.200 ± 0.022^{c,d}
Hexanoic acid	2.739 ± 0.197^{a}	0.597 ± 0.084^{b}	4.413 ± 0.324^{c}	3.289 ± 0.098^{a}	2.910 ± 0.287^{a}
Benzoic acid	0.116 ± 0.006^{a}	0.039 ± 0.005^{b}	0.058 ± 0.001^{b}	0.132 ± 0.002^{a}	0.079 ± 0.011^{c}
n-Decanoic acid	0.290 ± 0.041^{a}	7.320 ± 0.438^{b}	0.358 ± 0.051^{a}	7.054 ± 0.829^{b}	35.369 ± 3.933^{c}
Butanoic acid	0.695 ± 0.047^{a,d}	0.349 ± 0.006^{b}	0.722 ± 0.017^{a}	0.858 ± 0.010^{c}	0.585 ± 0.045^{d}
Total	4.751 ± 0.368	8.543 ± 0.545	6.241 ± 0.423	12.067 ± 0.950	39.595 ± 4.344

% Acids	0.02	0.00	0.01	0.02	0.05
**Esters**					
Ethyl 9-decenoate	0.377 ± 0.032^{a}	0.139 ± 0.003^{b}	0.295 ± 0.024^{a,c}	0.674 ± 0.012^{d}	0.236 ± 0.008^{b,c}
Ethyl decanoate	0.725 ± 0.053^{a}	0.305 ± 0.014^{b}	0.935 ± 0.032^{a,d}	1.635 ± 0.038^{c}	1.132 ± 0.098^{d}
Ethyl-2-phenyl acetate	0.078 ± 0.004^{a}	0.502 ± 0.055^{b}	1.205 ± 0.139^{c}	0.081 ± 0.011^{a}	1.385 ± 0.188^{c}
Diethyl succinate	0.474 ± 0.024^{a}	0.316 ± 0.010^{b,d}	0.530 ± 0.015^{a}	0.790 ± 0.000^{c}	0.433 ± 0.034^{a,d}
Ethyl-3-hydroxy butanoate	0.791 ± 0.063^{a}	0.315 ± 0.001^{b}	0.439 ± 0.023^{c}	1.801 ± 0.029^{d}	0.866 ± 0.033^{a,b}
Phenetyl acetate	0.481 ± 0.014^{a}	0.220 ± 0.018^{b}	1.181 ± 0.078^{c}	0.780 ± 0.022^{d}	0.805 ± 0.000^{d}
Ethyl octanoate	0.258 ± 0.020^{a}	0.040 ± 0.006^{b}	0.258 ± 0.010^{a}	0.277 ± 0.004^{a}	0.289 ± 0.008^{a}
Ethyl octadecanoate	0.102 ± 0.009^{a}	0.021 ± 0.003^{b}	0.124 ± 0.002^{c}	0.101 ± 0.000^{a}	0.039 ± 0.001^{b}
Isoamil laurate	0.168 ± 0.010^{a}	0.044 ± 0.006^{b}	0.107 ± 0.009^{c}	0.250 ± 0.002^{d}	0.086 ± 0.012^{c}
2-propenyl benzoate	0.162 ± 0.010^{a}	0.088 ± 0.003^{b}	0.115 ± 0.010^{b}	0.186 ± 0.001^{a}	0.166 ± 0.012^{a}
Ethyl octanoate	0.712 ± 0.057^{a}	0.324 ± 0.001^{b}	0.374 ± 0.053^{b}	0.754 ± 0.090^{a}	1.182 ± 0.064^{c}
Isoamyl acetate	59.939 ± 1.847^{a}	78.085 ± 2.202^{b}	48.711 ± 0.893^{a,c}	46.195 ± 0.066^{c}	59.892 ± 0.312^{a}
Ethyl nonanoate	0.054 ± 0.003^{a}	0.046 ± 0.006^{a}	0.080 ± 0.001^{b}	0.092 ± 0.003^{b,c}	0.097 ± 0.007^{c}
Ethyl lactacte	0.117 ± 0.006^{a}	0.137 ± 0.001^{a}	0.134 ± 0.011^{a}	0.129 ± 0.001^{a}	0.178 ± 0.004^{b}
Isoamyl lactate	0.045 ± 0.002^{a}	0.030 ± 0.004^{a}	0.092 ± 0.001^{b}	0.115 ± 0.002^{b}	0.161 ± 0.002^{c}
Ethyl acetate	50.234 ± 3.468^{a}	54.589 ± 2.479^{a}	55.946 ± 3.478^{a}	81.379 ± 22.289^{b}	100.025 ± 8.364^{c}
Total	114.717 ± 5.623	135.202 ± 4.813	110.524 ± 4.778	135.239 ± 12.570	166.971 ± 9.145
% Esters	0.20	0.22	0.16	0.23	0.21
**Aldehydes**					
Valeraldehyde	0.017 ± 0.002^{a}	0.077 ± 0.006^{b}	0.037 ± 0.005^{c}	0.075 ± 0.001^{b}	0.021 ± 0.003^{a}
Benzeneacetaldehyde	0.089 ± 0.004^{a}	0.137 ± 0.009^{b}	0.107 ± 0.004^{a}	0.174 ± 0.003^{c}	0.171 ± 0.004^{c}
2-Methyl valeraldehyde	0.721 ± 0.049^{a}	0.254 ± 0.021^{b}	0.208 ± 0.022^{b}	0.614 ± 0.029^{a,c}	0.515 ± 0.059^{c}
Acetaldehyde	35.580 ± 1.389^{a}	41.398 ± 1.579^{b}	49.810 ± 1.678^{c}	50.213 ± 2.623^{c}	62.314 ± 1.498^{d}
Total	36.406 ± 1.444	41.866 ± 1.616	50.162 ± 1.709	51.076 ± 2.656	63.021 ± 1.563
% Aldehydes	0.06	0.07	0.07	0.09	0.08
**C6 Alcohols**					
1-Hexanol	0.279 ± 0.010^{a}	0.274 ± 0.002^{a}	0.285 ± 0.028^{a}	0.304 ± 0.000^{a,b}	0.353 ± 0.012^{b}
(*Z*)-3-hexen-1-ol	1.050 ± 0.065^{a}	0.497 ± 0.014^{b}	0.528 ± 0.065^{b}	1.254 ± 0.023^{c}	0.239 ± 0.016^{d}
Total	1.330 ± 0.075	0.771 ± 0.016	0.814 ± 0.092	1.558 ± 0.023	0.592 ± 0.028
% C6 Alcohols	0.00	0.00	0.00	0.00	0.00

**Thiols**					
3-Methyl-thiol-1-Propanol	0.033 ± 0.004^{a}	0.074 ± 0.004^{a}	0.159 ± 0.015^{b}	0.154 ± 0.003^{b}	0.185 ± 0.007^{b}
% Thiols	0.00	0.00	0.00	0.00	0.00
**Phenols**					
Guaiacol	0.080 ± 0.009^{a}	0.036 ± 0.005^{a,c}	0.343 ± 0.005^{b}	0.000 ± 0.000^{c}	0.286 ± 0.033^{d}
Aceto-vanillone	0.190 ± 0.011^{a,c}	0.029 ± 0.004^{b}	0.158 ± 0.001^{a}	0.214 ± 0.010^{c}	0.110 ± 0.016^{d}
4-Hydroxy-Benzeneethanol	14.450 ± 0.965^{a}	1.492 ± 0.211^{b}	6.888 ± 0.107^{c}	10.737 ± 0.106^{d}	5.330 ± 0.754^{c}
4-vinyl-guayacol	0.371 ± 0.034^{a}	0.275 ± 0.016^{a}	0.991 ± 0.044^{b}	1.015 ± 0.017^{b}	0.781 ± 0.073^{c}
2,6-Dimethoxy-phenol	0.164 ± 0.018^{a}	0.120 ± 0.014^{b}	0.130 ± 0.009^{b}	0.169 ± 0.009^{a}	0.113 ± 0.008^{b}
Total	15.254 ± 1.336	1.952 ± 0.250	8.510 ± 0.167	12.135 ± 0.141	6.620 ± 0.884
% Phenols	0.03	0.00	0.01	0.02	0.01
**Terpenes**					
Linalool	0.064 ± 0.002^{a}	0.089 ± 0.006^{b}	0.120 ± 0.006^{c,d,e}	0.113 ± 0.001^{d}	0.137 ± 0.001^{e}
% Terpenes	0.00	0.00	0.00	0.00	0.00
**Lactones**					
2,3-Dihydro-Benzofuran	0.214 ± 0.011^{a}	0.280 ± 0.018^{a,c}	0.454 ± 0.016^{b}	0.279 ± 0.020^{a,c}	0.336 ± 0.003^{c}
% Lactones	0.00	0.00	0.00	0.00	0.00

SD48h: sun-dried grapes during 48h. SD96h: sun-dried grapes during 96h. CH48h: climatic chamber drying during 48h. CH96h: climatic chamber drying during 96h. Different letters in superscript mean significant differences ($p < 0.05$) between samples determined by a two-way ANOVA according to Bonferroni's multiple range (BSD) test.

Table S2. Volatile compound concentration (μg/L) in wines elaborated with GS presence during 2018 vintage.

	**Control**	**SD48h**	**SD96h**	**CH48h**	**CH96h**
**Methanol**	13527.900 ± 5.900^{a}	11998.200 ± 5.182^{a}	16894.600 ± 2.826^{a}	14230.100 ± 3.617^{a}	19856.300 ± 3.140^{a}
% Methanol	22.01	38.06	45.55	60.19	39.61
**Higher alcohols**					
2-Methyl-1-Propanol	41.000 ± 0.041^{a}	0.441 ± 0.028^{a}	0.063 ± 0.001^{b}	0.429 ± 0.005^{a}	0.406 ± 0.043^{a}
2-Propanol	48996.300 ± 29.634^{a}	50046.5 ± 18.468^{a}	59897.537 ± 58.945^{b}	48798.700 ± 46.923^{a}	65936.594 ± 109.459^{b}
Total	49037.300 ± 29.675	50046.900 ± 18.496	59897.600 ± 58.946	48799.100 ± 46.928	65937.000 ± 109.502
% Higher alcohols	77.48	61.05	53.69	38.21	59.67
**Alcohols**					
3-Penten-2-ol	0.633 ± 0.086^{a,c,d}	1.550 ± 0.046^{b}	0.086 ± 0.003^{a,d}	0.899 ± 0.058^{c}	0.049 ± 0.040^{d}
1-Nonanol	0.590 ± 0.014^{a,c}	0.044 ± 0.064^{b}	0.708 ± 0.006^{a}	0.261 ± 0.080^{c,b}	0.639 ± 0.056^{a}
Benzyl alcohol	1.031 ± 0.016^{a}	0.126 ± 0.023^{b}	1.289 ± 0.026^{a}	0.382 ± 0.028^{b}	1.230 ± 0.078^{a}
2-Phenylethanol	77.853 ± 2.225^{a}	29.600 ± 2.695^{b,c}	17.865 ± 2.362^{b}	58.256 ± 1.204^{c}	57.302 ± 3.435^{c}
3-Ethoxy-1-Propanol	0.190 ± 0.016^{a}	0.125 ± 0.015^{a}	0.065 ± 0.004^{b}	0.060 ± 0.019^{b}	0.063 ± 0.031^{b}

2-Nonanol	3.824 ± 1.348^{a,b}	3.279 ± 0.060^{a}	4.379 ± 0.106^{b}	1.532 ± 0.075^{c}	4.236 ± 0.108^{b}
DL-2,3-Butanediol	6.715 ± 0.293^{a}	3.497 ± 0.037^{b}	4.705 ± 0.114^{a,b}	4.249 ± 0.047^{a,b}	4.735 ± 0.272^{a,b}
3-Ethyl-2-Pentanol	1.362 ± 0.004^{a}	0.257 ± 0.073^{b}	1.166 ± 0.024^{a}	0.746 ± 0.091^{a,b}	1.306 ± 0.094^{a}
3-Methyl-1-Pentanol	0.437 ± 0.021^{a}	0.277 ± 0.018^{a,b}	0.171 ± 0.022^{b}	0.264 ± 0.022^{a,b}	0.252 ± 0.010^{a,b}
4-Methyl-1-Pentanol	0.531 ± 0.023^{a}	0.102 ± 0.020^{b}	0.282 ± 0.003^{a,b}	0.239 ± 0.025^{b,c}	0.378 ± 0.018^{a,c}
1-Octanol	0.273 ± 0.003^{a}	0.037 ± 0.012^{b}	0.169 ± 0.018^{a,b}	0.110 ± 0.016^{b}	0.287 ± 0.011^{a}
Total	93.439 ± 4.048	38.893 ± 3.063	30.884 ± 2.688	66.999 ± 1.664	70.478 ± 4.154
% Alcohols	0.2	0.1	0.1	0.3	0.1
**Acids**					
Heptanoic acid	0.105 ± 0.081^{a}	1.254 ± 0.036^{b}	1.092 ± 0.011^{b}	0.365 ± 0.101^{b,c}	0.872 ± 0.071^{b,c}
2-Hexenoic acid	0.249 ± 0.073^{a}	0.993 ± 0.036^{b}	1.211 ± 0.024^{b}	0.081 ± 0.091^{a}	0.968 ± 0.218^{b}
3-Methylbutanoic acid	0.103 ± 0.014^{a}	0.974 ± 0.084^{b}	0.280 ± 0.013^{a}	0.179 ± 0.017^{a}	0.313 ± 0.012^{a}
Hexanoic acid	0.925 ± 0.063^{a}	2.323 ± 0.262^{a,b}	1.290 ± 0.021^{a}	1.220 ± 0.079^{a}	3.592 ± 0.056^{b}
Benzoic acid	0.102 ± 0.002^{a}	0.096 ± 0.017^{a}	0.107 ± 0.003^{a}	0.142 ± 0.021^{a}	0.143 ± 0.015^{a}
n-Decanoic acid	1.346 ± 0.158^{a}	0.384 ± 0.006^{b}	0.646 ± 0.016^{b}	0.432 ± 0.007^{b}	0.369 ± 0.032^{b}
Butanoic acid	1.819 ± 0.062^{a}	0.280 ± 0.017^{b}	1.097 ± 0.002^{a,b}	0.703 ± 0.021^{b}	1.258 ± 0.669^{a}
Total	4.649 ± 0.453	6.304 ± 0.458	5.723 ± 0.089	3.122 ± 0.338	7.515 ± 1.073
% Acids	0.00	0.00	0.00	0.00	0.00
**Esters**					
Ethyl 9-decenoate	1.264 ± 0.027^{a}	0.124 ± 0.003^{b}	0.209 ± 0.014^{b}	0.449 ± 0.004^{b}	0.477 ± 0.003^{b}
Ethyl decanoate	2.239 ± 0.256^{a,c}	0.195 ± 0.050^{b}	3.874 ± 0.069^{a}	1.230 ± 0.063^{c,b}	3.305 ± 0.442^{a}
Ethyl-2-phenyl acetate	0.254 ± 0.013^{a}	0.780 ± 0.069^{a}	2.807 ± 0.028^{b}	0.131 ± 0.086^{a}	0.135 ± 0.061^{a}
Diethyl succinate	1.950 ± 0.238^{a}	0.532 ± 0.011^{b}	1.527 ± 0.002^{a}	1.561 ± 0.014^{a}	1.903 ± 0.096^{a}
Ethyl-3-hydroxy butanoate	0.840 ± 0.086^{a,b}	0.236 ± 0.035^{a}	1.755 ± 0.024^{b}	0.727 ± 0.043^{a,b}	2.361 ± 0.303^{b}
Phenetyl acetate	1.172 ± 0.127^{a,c}	0.165 ± 0.098^{b}	1.055 ± 0.040^{a,b}	0.547 ± 0.022^{a,b}	2.065 ± 0.859^{c}
Ethyl octanoate	0.086 ± 0.005^{a}	0.097 ± 0.018^{a}	0.345 ± 0.008^{b}	0.225 ± 0.022^{a,b}	0.344 ± 0.015^{b}
Ethyl octadecanoate	0.436 ± 0.038^{a}	0.105 ± 0.004^{b}	0.288 ± 0.007^{a,b}	0.127 ± 0.005^{b}	0.153 ± 0.003^{b}
Isoamil laurate	0.375 ± 0.039^{a}	0.113 ± 0.006^{b}	0.112 ± 0.003^{b}	0.184 ± 0.007^{b}	0.129 ± 0.005^{b}
2-propenyl benzoate	0.110 ± 0.003^{a}	0.470 ± 0.013^{b}	0.107 ± 0.003^{a}	0.227 ± 0.017^{a}	0.274 ± 0.012^{a,b}
Ethyl octanoate	0.518 ± 0.006^{a,b}	0.315 ± 0.071^{a}	0.546 ± 0.038^{a,b}	1.061 ± 0.089^{c}	1.005 ± 0.062^{b,c}
Isoamyl acetate	63.693 ± 6.299^{a,b}	119.644 ± 6.936^{a}	80.223 ± 1.688^{a,b}	103.628 ± 8.668^{a,b}	48.684 ± 6.091^{b}
Ethyl nonanoate	0.188 ± 0.013^{a}	0.027 ± 0.011^{b}	0.197 ± 0.001^{a}	0.088 ± 0.014^{b}	0.185 ± 0.100^{a}
Ethyl lactacte	1.324 ± 0.015^{a}	0.272 ± 0.075^{b}	0.514 ± 0.006^{b}	0.406 ± 0.094^{b}	0.564 ± 0.066^{b}
Isoamyl lactate	0.292 ± 0.008^{a}	0.023 ± 0.034^{b}	0.358 ± 0.004^{a}	0.090 ± 0.043^{b}	0.335 ± 0.300^{a}
Ethyl acetate	79.650 ± 6.167^{a,b}	42.398 ± 3.429^{a}	59.210 ± 0.235^{a,c}	110.258 ± 3.863^{b,c}	119.087 ± 3.354^{b}
Total	154.391 ± 13.342	165.497 ± 10.863	153.127 ± 2.169	181.006 ± 11.773	220.939 ± 13.054
% Esters	0.25	0.52	0.41	0.93	0.36
**Aldehydes**					
Valeraldehyde	0.605 ± 0.069^{a}	0.249 ± 0.088^{b,c}	0.385 ± 0.002^{a,c}	0.033 ± 0.001^{b}	0.696 ± 0.096^{a}
Benzeneacetaldehyde	0.613 ± 0.021^{a}	0.460 ± 0.094^{a}	0.498 ± 0.009^{a}	0.101 ± 0.012^{b}	0.435 ± 0.043^{a}

2-Methyl valeraldehyde	0.432	±	0.034^{a}	0.177	±	0.026^{b,c}	0.061	±	0.004^{b}	0.336	±	0.033^{a,c}	0.458	±	0.023^{a}
Acetaldehyde	39.874	±	3.783^{a}	69.879	±	2.424^{a,b}	79.846	±	5.798^{a,b}	75.812	±	3.032^{a,b}	89.645	±	2.633^{b}
Total	41.523	±	3.906	70.765	±	2.633	80.791	±	5.814	76.282	±	3.078	91.233	±	2.794
% Aldehydes	0.07			0.22			0.22			0.32			0.18		
**C6 Alcohols**															
1-Hexanol	0.465	±	0.026^{a}	0.644	±	0.022^{a,b}	0.352	±	0.034^{a}	0.921	±	0.027^{b}	0.644	±	0.019^{a,b}
(*Z*)-3-hexen-1-ol	1.043	±	0.050^{a,b}	0.414	±	0.060^{a}	1.055	±	0.028^{a,b}	0.914	±	0.075^{a,b}	1.493	±	0.052^{b}
Total	1.508	±	0.050	1.058	±	0.060	1.407	±	0.028	1.835	±	0.075	2.137	±	0.052
% C6 Alcohols	0.00			0.00			0.00			0.01			0.00		
**Thiols**															
3-Methylthiol-1-propanol	0.040	±	0.039^{a}	0.180	±	0.032^{b}	0.234	±	0.004^{a,b}	0.131	±	0.040^{b}	0.319	±	0.028^{a,b}
% Thiols	0.00			0.00			0.00			0.00			0.00		
**Phenols**															
Guaiacol	1.039	±	0.047^{a}	0.163	±	0.041^{b}	0.448	±	0.004^{b}	0.273	±	0.051^{b}	0.422	±	0.036^{b}
Acetovanillone	0.417	±	0.046^{a}	0.058	±	0.013^{b}	0.237	±	0.006^{a,b}	0.142	±	0.016^{b}	0.263	±	0.011^{a}
4-Hydroxy-Benzeneethanol	7.470	±	0.560^{a}	1.925	±	0.318^{b}	2.274	±	0.055^{b,c}	4.545	±	0.398^{a,b}	5.582	±	0.279^{a,c}
4-vinylguayacol	1.212	±	0.074^{a}	0.608	±	0.075^{a}	5.092	±	0.048^{b}	2.053	±	0.094^{a}	2.260	±	0.066^{a}
2,6-Dimethoxyphenol	0.762	±	0.074^{a}	0.124	±	0.054^{b}	0.540	±	0.005^{a,c}	0.192	±	0.067^{b,c}	0.497	±	0.047^{a,c}
Total	10.900	±	0.802	2.877	±	0.500	8.592	±	0.117	7.205	±	0.625	9.024	±	0.087
% Phenols	0.02			0.01			0.02			0.03			0.02		
**Terpenes**															
Linalool	0.104	±	0.030^{a}	0.163	±	0.024^{a}	0.307	±	0.006^{a,b}	0.199	±	0.014^{b}	0.468	±	0.006^{c}
% Terpenes	0.00			0.00			0.00			0.00			0.00		
**Lactones**															
2,3-Dihydro-Benzofuran	0.201	±	0.010^{a}	0.378	±	0.012^{a}	1.215	±	0.029^{b}	0.578	±	0.052^{a,c}	1.039	±	0.086^{b,c}
% Lactones	0.00			0.00			0.00			0.00			0.00		

SD48h: sun-dried grapes during 48h. SD96h: sun-dried grapes during 96h. CH48h: climatic chamber drying during 48h. CH96h: climatic chamber drying during 96h. Different letters in superscript mean significant differences ($p < 0.05$) between samples determined by a two-way ANOVA according to Bonferroni's multiple range (BSD) test.

Table S3. Volatile compound concentration (μg/L) in wines elaborated without GS during 2019 vintage.

	Control			SD48h			SD96h			CH48h			CH96h		
**Methanol**	7896.500	±	4.727^{a}	10539.800	±	15.931^{b}	11035.000	±	2.483^{b}	10987.400	±	2.898^{b}	11138.900	±	3.271^{b}
% Methanol	41.81			50.07			47.99			39.13			38.69		
**Higher alcohols**															
2-Methyl-1-Propanol	6.000	±	0.001^{a}	1.000	±	0.021^{b}	1.200	±	0.002^{b}	1.700	±	0.001^{b}	2.700	±	0.004^{c}
2-Propanol	10873.800	±	104.082^{a}	9999.870	±	84.318^{a}	11563.680	±	82.221^{a}	16879.450	±	72.788^{b}	17201.030	±	82.163^{b}
Total	10879.800	±	104.082	10000.870	±	84.318	11564.880	±	82.223	16881.150	±	72.789	17203.730	±	82.167

% Higher alcohols	57.60	47.51	50.30	60.12	59.76
**Alcohols**					
3-Penten-2-ol	0.021 ± 0.003 [a]	0.120 ± 0.017 [a]	2.977 ± 0.420 [b]	0.013 ± 0.001 [a]	0.082 ± 0.008 [a]
1-Nonanol	0.188 ± 0.011 [a]	54.282 ± 7.249 [b]	15.019 ± 2.044 [c]	0.829 ± 0.053 [a]	3.845 ± 0.432 [a]
Benzyl alcohol	0.153 ± 0.020 [a]	1.206 ± 15.134 [b]	9.869 ± 1.310 [a]	1.676 ± 0.115 [a]	9.315 ± 1.050 [a]
2-Phenylethanol	17.540 ± 0.106 [a]	45.350 ± 2.309 [b]	18.492 ± 0.989 [a]	17.829 ± 1.095 [a]	68.447 ± 4.344 [c]
3-Ethoxy-1-Propanol	0.049 ± 0.000 [a]	0.180 ± 0.025 [b]	0.035 ± 0.005 [a]	0.096 ± 0.009 [c]	0.153 ± 0.005 [b]
2-Nonanol	1.067 ± 0.031 [a]	1.609 ± 0.293 [a]	1.754 ± 0.940 [a]	5.267 ± 0.658 [b]	3.148 ± 0.199 [b]
DL-2,3-Butanediol	0.371 ± 0.010 [a]	4.047 ± 0.858 [b]	7.858 ± 0.569 [c]	7.291 ± 0.567 [c]	4.692 ± 0.371 [b]
3-Ethyl-2-Pentanol	0.275 ± 0.009 [a]	10.020 ± 1.007 [b]	6.664 ± 0.852 [c]	1.159 ± 0.031 [a]	2.595 ± 0.163 [a]
3-Methyl-1-Pentanol	0.075 ± 0.003 [a]	1.787 ± 0.139 [b]	2.392 ± 0.322 [c]	0.207 ± 0.006 [a]	0.604 ± 0.040 [a]
4-Methyl-1-Pentanol	0.106 ± 0.002 [a]	3.872 ± 0.394 [b]	1.120 ± 0.128 [c]	0.308 ± 0.011 [a]	0.962 ± 0.071 [a]
1-Octanol	0.031 ± 0.001 [a]	0.283 ± 0.040 [b]	0.120 ± 0.007 [a]	0.269 ± 0.016 [b]	0.293 ± 0.005 [b]
Total	19.875 ± 0.196	122.757 ± 27.645	66.180 ± 52.578	34.944 ± 3.562	146.136 ± 15.687
% Alcohols	0.11	1.09	0.29	0.12	0.51
**Acids**					
Heptanoic acid	0.384 ± 0.036 [a]	4.177 ± 0.693 [b]	0.143 ± 0.020 [a]	1.029 ± 0.013 [a]	3.935 ± 0.448 [b]
2-Hexenoic acid	0.255 ± 0.009 [a]	16.218 ± 0.957 [b]	9.086 ± 1.266 [c]	0.789 ± 0.025 [a]	10.821 ± 1.463 [c]
3-Methyl-butanoic acid	0.111 ± 0.003 [a]	7.411 ± 0.608 [b]	10.046 ± 1.378 [c]	0.455 ± 0.006 [a]	2.516 ± 0.219 [d]
Hexanoic acid	1.316 ± 0.004 [a]	11.832 ± 6.081 [b]	1.885 ± 0.267 [a]	2.320 ± 0.118 [a,c]	4.722 ± 0.611 [c]
Benzoic acid	0.082 ± 0.002 [a]	3.605 ± 0.059 [b]	4.823 ± 0.671 [c]	0.303 ± 0.012 [a]	1.515 ± 0.167 [d]
n-Decanoic acid	0.487 ± 0.013 [a]	3.439 ± 0.486 [a,c]	48.619 ± 6.736 [b]	2.565 ± 0.276 [a,c]	11.780 ± 0.787 [c]
Butanoic acid	0.388 ± 0.029 [a]	8.785 ± 0.678 [b]	9.421 ± 1.277 [b]	1.010 ± 0.035 [a]	3.197 ± 0.361 [c]
Total	3.024 ± 0.096	55.468 ± 9.564	84.023 ± 11.614	8.471 ± 0.485	38.486 ± 4.057
% Acids	0.02	0.26	0.37	0.03	0.13
**Esters**					
Ethyl 9-decenoate	0.227 ± 0.021 [a]	2.572 ± 0.364 [b]	3.652 ± 0.028 [c]	2.061 ± 0.153 [b]	3.242 ± 0.118 [c]
Ethyl decanoate	0.554 ± 0.015 [a]	2.760 ± 0.237 [b]	1.076 ± 0.086 [c]	5.218 ± 0.382 [d]	5.160 ± 2.759 [d]
Ethyl-2-phenyl acetate	1.166 ± 0.134 [a]	1.772 ± 0.373 [b]	1.273 ± 0.285 [a]	0.183 ± 0.006 [c]	1.918 ± 0.113 [b]
Diethyl succinate	0.249 ± 0.020 [a]	0.164 ± 0.023 [a]	0.075 ± 0.011 [a]	0.223 ± 0.013 [a]	1.163 ± 0.241 [b]
Ethyl-3-hydroxy butanoate	0.422 ± 0.014 [a]	4.974 ± 0.032 [b]	2.015 ± 0.052 [a]	3.658 ± 0.026 [b]	11.141 ± 0.758 [c]
Phenetyl acetate	0.092 ± 0.003 [a]	1.445 ± 0.086 [b,c]	1.853 ± 0.060 [b]	3.238 ± 0.240 [d]	1.138 ± 0.031 [c]
Ethyl octanoate	0.121 ± 0.006 [a]	2.679 ± 0.099 [b]	8.425 ± 1.171 [c]	0.523 ± 0.041 [a]	3.421 ± 0.398 [b]

Ethyl octadecanoate	0.069	±	0.005[a]	4.123	±	0.558[b]	4.129	±	0.573[b]	0.133	±	0.007[a]	3.262	±	0.422[c]
Isoamil laurate	0.053	±	0.006[a]	0.230	±	0.033[a]	4.178	±	0.580[b]	0.202	±	0.015[a]	1.044	±	0.114[a]
2-propenyl benzoate	0.059	±	0.001[a]	0.269	±	0.038[a]	4.516	±	0.635[b]	0.306	±	0.009[a]	4.001	±	0.535[b]
Ethyl octanoate	0.683	±	0.015[a]	4.718	±	0.059[b]	7.027	±	0.016[c]	3.004	±	0.425[d]	6.050	±	0.484[b,c]
Isoamyl acetate	6.283	±	0.795[a]	2.252	±	0.100[b,c]	3.311	±	0.172[b]	1.747	±	0.147[c]	2.701	±	0.045[b]
Ethyl nonanoate	0.088	±	0.004[a]	4.434	±	0.080[b]	1.942	±	0.249[c]	0.340	±	0.019[a]	0.826	±	0.057[a]
Ethyl lactacte	0.065	±	0.008[a]	0.856	±	0.098[b]	0.713	±	0.097[b]	0.109	±	0.015[a]	0.771	±	0.073[b]
Isoamyl lactate	0.053	±	0.002[a]	2.670	±	0.080[b]	0.009	±	0.001[a]	0.436	±	0.031[a]	2.011	±	0.205[c]
Ethyl acetate	45.821	±	1.760[a,b]	39.869	±	7.059[a]	32.358	±	0.206[a,b]	59.374	±	3.096[b]	84.127	±	3.495[c]
Total	56.004	±	2.808	75.786	±	9.317	76.553	±	3.918	80.753	±	4.626	131.976	±	9.847
% Esters	0.30			0.36			0.35			0.29			0.46		
**Aldehydes**															
Valeraldehyde	0.060	±	0.005[a]	0.694	±	0.071[b]	0.075	±	0.011[a,c]	0.209	±	0.018[c]	0.130	±	0.008[c]
Benzeneacetaldehyde	0.110	±	0.003[a]	0.822	±	0.084[b]	0.098	±	0.014[a]	0.719	±	0.057[b]	0.808	±	0.055[b]
2-Methyl valeraldehyde	0.115	±	0.007[a]	2.249	±	0.061[b]	3.097	±	0.404[c]	0.367	±	0.028[a]	1.074	±	0.051[d]
Acetaldehyde	28.743	±	3.031[a]	45.168	±	2.775[b]	49.896	±	5.095[b]	71.685	±	2.430[c]	80.268	±	2.743[c]
Total	29.027	±	3.046	48.933	±	2.991	53.166	±	5.524	72.980	±	2.532	82.281	±	2.849
% Aldehydes	0.15			0.23			0.23			0.26			0.29		
**C6 Alcohols**															
1-Hexanol	0.084	±	0.006[a]	1.277	±	0.034[b]	0.587	±	0.034[c]	0.243	±	0.006[a]	0.070	±	0.010[a]
(*Z*)-3-hexen-1-ol	0.607	±	0.012[a]	1.294	±	8.200[b]	1.719	±	2.278[c]	1.518	±	0.032[a]	1.749	±	0.030[a]
Total	0.690	±	0.012	2.571	±	8.200	2.306	±	2.278	1.762	±	0.032	1.819	±	0.040
% C6 Alcohols	0.00			0.01			0.01			0.01			0.01		
**Thiols**															
3-Methyl-thiol-1-Propanol	0.030	±	0.001[a]	0.080	±	0.011[a]	0.638	±	0.090[b]	0.171	±	0.007[a]	0.927	±	0.101[c]
% Thiols	0.00			0.00			0.00			0.00			0.00		
**Phenols**															
Guaiacol	0.181	±	0.016[a]	1.661	±	0.235[b]	1.856	±	0.478[b]	0.234	±	0.019[c]	0.686	±	0.094[d]
Acetovanillone	0.051	±	0.004[a]	0.255	±	0.036[a]	3.365	±	0.463[b]	1.029	±	0.131[c]	1.663	±	0.185[c]
4-Hydroxy-Benzeneethanol	1.870	±	0.056[a]	13.972	±	1.976[b]	17.334	±	1.146[c]	15.834	±	0.381[d]	22.121	±	1.279[e]
4-vinyl-guayacol	0.179	±	0.000[a]	3.603	±	0.061[b]	11.544	±	1.611[c]	1.712	±	0.048[a]	8.117	±	0.943[c]

2,6-Dimethoxyphenol	0.071 ± 0.002^{a}	0.913 ± 0.129^{a}	6.705 ± 0.918^{b}	0.712 ± 0.052^{a}	2.702 ± 0.331^{c}
Total	2.352 ± 0.078	20.404 ± 6.937	40.805 ± 23.616	9.522 ± 0.632	35.289 ± 38.832
% Phenols	0.01	0.10	0.18	0.03	0.12
**Terpenes**					
Linalool	0.070 ± 0.002^{a}	0.085 ± 0.012^{a}	0.523 ± 0.074^{b}	0.349 ± 0.025^{a}	1.105 ± 0.173^{c}
% Terpenes	0.00	0.00	0.00	0.00	0.02
**Lactones**					
2,3-Dihydro-Benzofuran	0.308 ± 0.022^{a}	0.358 ± 0.056^{a}	0.596 ± 0.057^{b}	0.794 ± 0.100^{c}	1.060 ± 0.188^{c}
% Lactones	0.00	0.01	0.09	0.00	0.02

SD48h: sun-dried grapes during 48h. SD96h: sun-dried grapes during 96h. CH48h: climatic chamber drying during 48h. CH96h: climatic chamber drying during 96h. Different letters in superscript mean significant differences ($p < 0.05$) between samples determined by a two-way ANOVA according to Bonferroni's multiple range (BSD) test.

Table S4. Volatile compound concentration (μg/L) in wines elaborated with GS presence during 2019 vintage.

	**Control**	**SD48h**	**SD96h**	**CH48h**	**CH96h**
**Methanol**	14001.600 ± 4.148^{a}	10552.700 ± 4.567^{b}	12054.900 ± 10.005^{c}	11025.900 ± 2.718^{d}	18000.300 ± 3.239^{c}
% Methanol	18.94	21.87	14.77	18.93	19.03
**Higher alcohols**					
2-Methyl-1-Propanol	98.530 ± 0.002^{a}	1.100 ± 0.000^{b}	2.500 ± 0.001^{b}	3.900 ± 0.012^{b}	8.700 ± 0.006^{b}
2-Propanol	59636.300 ± 91.316^{a}	37524.200 ± 64.925^{b}	69374.800 ± 100.264^{c}	46890.200 ± 89.988^{d}	76277.800 ± 81.357^{e}
Total	59734.830 ± 91.317	37525.300 ± 64.925	69327.300 ± 100.265	46894.100 ± 90.000	76286.500 ± 81.363
% Higher alcohols	80.81	77.77	84.96	80.52	80.64
**Alcohols**					
3-Penten-2-ol	3.984 ± 0.001^{a}	0.043 ± 0.000^{b}	0.029 ± 0.001^{b}	0.193 ± 0.003^{b}	0.054 ± 0.004^{b}
1-Nonanol	1.134 ± 0.030^{a}	0.435 ± 0.033^{b}	0.592 ± 0.024^{c}	2.198 ± 0.024^{d}	0.865 ± 0.066^{e}
Benzyl alcohol	0.516 ± 0.053^{a}	0.613 ± 0.087^{a}	1.051 ± 0.042^{b}	0.528 ± 0.092^{a}	1.800 ± 0.054^{c}
2-Phenylethanol	38.883 ± 0.654^{a}	31.506 ± 0.758^{b}	36.604 ± 0.524^{a}	54.934 ± 4.136^{c}	30.391 ± 0.608^{b}
3-Ethoxy-1-Propanol	2.880 ± 0.010^{a}	0.014 ± 0.002^{b}	0.040 ± 0.002^{b}	1.090 ± 0.027^{c}	0.042 ± 0.006^{b}
2-Nonanol	1.485 ± 0.117^{a}	8.812 ± 0.542^{b}	1.759 ± 0.094^{a}	2.765 ± 0.071^{c}	6.502 ± 0.162^{d}
DL-2,3-Butanediol	2.286 ± 0.038^{a}	2.751 ± 0.039^{a}	0.812 ± 0.030^{b}	2.319 ± 0.049^{a}	7.010 ± 0.194^{c}
3-Ethyl-2-Pentanol	9.619 ± 0.072^{a}	0.624 ± 0.052^{b}	1.351 ± 0.058^{c}	2.063 ± 0.057^{d}	1.823 ± 0.020^{c,d}
3-Methyl-1-Pentanol	1.604 ± 0.027^{a}	0.190 ± 0.006^{b}	0.355 ± 0.021^{b}	0.535 ± 0.043^{b}	0.421 ± 0.021^{b}
4-Methyl-1-Pentanol	2.291 ± 0.019^{a}	0.180 ± 0.011^{b}	0.360 ± 0.015^{c}	0.554 ± 0.045^{d}	0.495 ± 0.010^{c,d}
1-Octanol	1.077 ± 0.006^{a}	0.139 ± 0.012^{b}	0.242 ± 0.005^{b}	0.676 ± 0.069^{b}	0.294 ± 0.016^{b}
Total	65.761 ± 1.026	45.307 ± 1.541	43.196 ± 0.816	67.855 ± 4.616	49.698 ± 1.161

% Alcohols	0.09	0.09	0.05	0.12	0.05
**Acids**					
Heptanoic acid	0.868 ± 0.001[a]	0.966 ± 0.064[b]	0.800 ± 0.001[a]	1.170 ± 0.034[c]	1.129 ± 0.124[c]
2-Hexenoic acid	0.378 ± 0.012[a]	0.900 ± 0.066[b]	0.240 ± 0.010[a]	1.593 ± 0.064[c]	2.074 ± 0.056[d]
3-Methyl-butanoic acid	1.314 ± 0.029[a]	3.271 ± 0.045[b]	0.533 ± 0.023[c]	1.015 ± 0.099[d]	0.651 ± 0.000[c]
Hexanoic acid	2.753 ± 0.125[a]	4.148 ± 0.154[b]	5.814 ± 0.100[c]	3.931 ± 0.043[b]	9.058 ± 0.056[d]
Benzoic acid	0.138 ± 0.006[a]	0.183 ± 0.003[b]	0.096 ± 0.004[c]	0.511 ± 0.049[d]	0.276 ± 0.019[e]
n-Decanoic acid	0.535 ± 0.099[a]	1.978 ± 0.076[b]	2.123 ± 0.080[b]	6.980 ± 0.099[c]	2.092 ± 0.029[b]
Butanoic acid	0.968 ± 0.013[a]	1.233 ± 0.076[a]	0.257 ± 0.011[b]	4.317 ± 0.055[c]	1.954 ± 0.076[d]
Total	6.953 ± 0.285	12.680 ± 0.484	9.862 ± 0.228	19.515 ± 0.442	17.235 ± 0.360
% Acids	0.01	0.03	0.01	0.03	0.02
**Esters**					
Ethyl 9-decenoate	1.276 ± 0.008[a]	1.071 ± 0.076[a]	0.179 ± 0.006[b]	5.218 ± 0.051[c]	0.557 ± 0.019[d]
Ethyl decanoate	0.802 ± 0.168[a]	1.793 ± 0.133[b]	3.262 ± 0.135[c]	5.230 ± 0.164[d]	5.297 ± 0.092[d]
Ethyl-2-phenyl acetate	0.556 ± 0.041[a]	0.135 ± 0.012[b]	3.829 ± 0.032[c]	1.181 ± 0.041[d]	0.106 ± 0.015[d]
Diethyl succinate	0.762 ± 0.004[a]	0.188 ± 0.010[b]	0.250 ± 0.003[b]	0.416 ± 0.021[c]	2.102 ± 0.066[d]
Ethyl-3-hydroxy butanoate	0.431 ± 0.107[a]	1.409 ± 0.083[b]	1.907 ± 0.085[c]	7.001 ± 0.114[d]	3.079 ± 0.048[e]
Phenetyl acetate	0.357 ± 0.022[a]	1.473 ± 0.014[b]	0.472 ± 0.018[a]	8.420 ± 0.255[c]	3.232 ± 0.103[d]
Ethyl octanoate	0.815 ± 0.031[a]	0.311 ± 0.018[b]	0.469 ± 0.025[c]	1.136 ± 0.014[d]	0.542 ± 0.038[c]
Ethyl octadecanoate	0.474 ± 0.010[a]	0.358 ± 0.022[b]	0.217 ± 0.008[c]	0.405 ± 0.045[d]	0.588 ± 0.020[e]
Isoamil laurate	0.010 ± 0.005[a]	0.073 ± 0.010[a,d]	0.116 ± 0.004[b,d]	1.274 ± 0.065[c]	0.179 ± 0.003[b]
2-propenyl benzoate	0.684 ± 0.002[a]	0.430 ± 0.015[b]	0.073 ± 0.001[c]	0.754 ± 0.067[d]	0.213 ± 0.009[e]
Ethyl octanoate	0.250 ± 0.012[a]	0.411 ± 0.043[b]	0.405 ± 0.009[b]	2.304 ± 0.084[c]	0.576 ± 0.023[d]
Isoamyl acetate	0.170 ± 0.022[a]	0.516 ± 0.031[b]	5.488 ± 0.338[c]	1.854 ± 0.020[d]	4.247 ± 0.056[e]
Ethyl nonanoate	0.618 ± 0.031[a]	0.338 ± 0.019[b]	0.429 ± 0.025[c]	0.991 ± 0.083[d]	0.352 ± 0.018[b]
Ethyl lactacte	0.435 ± 0.018[a]	0.097 ± 0.012[b]	0.106 ± 0.014[b]	0.141 ± 0.002[c]	0.211 ± 0.001[d]
Isoamyl lactate	0.464 ± 0.001[a]	0.203 ± 0.021[b]	0.360 ± 0.001[c]	1.217 ± 0.035[d]	0.551 ± 0.014[e]
Ethyl acetate	40.951 ± 3.441[a]	45.889 ± 2.435[a]	69.681 ± 0.451[b]	115.186 ± 0.226[c]	129.874 ± 3.460[d]
Total	69.054 ± 10.323	54.696 ± 5.954	87.244 ± 1.157	152.729 ± 1.287	151.705 ± 0.945
% Esters	0.09	0.11	0.11	0.26	0.16
**Aldehydes**					
Valeraldehyde	0.970 ± 0.017[a]	0.315 ± 0.002[b]	0.261 ± 0.013[b]	0.547 ± 0.050[c]	0.096 ± 0.014[d]

Benzeneacetaldehyde	0.763 ± 0.017^{a}	0.452 ± 0.012^{b}	0.394 ± 0.013^{b}	1.838 ± 0.017^{c}	0.824 ± 0.017^{a}
2-Methyl valeraldehyde	0.481 ± 0.009^{a}	0.209 ± 0.004^{b}	0.085 ± 0.007^{c}	0.802 ± 0.076^{d}	0.508 ± 0.046^{a}
Acetaldehyde	31.970 ± 2.659^{a}	53.036 ± 2.137^{b}	68.981 ± 1.440^{c}	63.101 ± 0.557^{d}	78.793 ± 2.716^{e}
Total	34.184 ± 2.702	54.012 ± 2.154	69.721 ± 1.474	66.288 ± 0.700	80.222 ± 2.792
% Aldehydes	0.05	0.11	0.09	0.11	0.08
**C6 Alcohols**					
1-Hexanol	0.110 ± 0.005^{a}	0.082 ± 0.003^{a}	0.548 ± 0.004^{b}	0.386 ± 0.006^{c}	0.215 ± 0.036^{d}
(*Z*)-3-hexen-1-ol	3.189 ± 0.002^{a}	1.215 ± 0.053^{b}	1.281 ± 0.001^{b}	2.852 ± 0.065^{c}	1.982 ± 0.079^{d}
Total	3.299 ± 0.002	1.297 ± 0.053	1.829 ± 0.001	3.067 ± 0.065	2.368 ± 0.079
% C6 Alcohols	0.00	0.00	0.00	0.00	0.00
**Thiols**					
3-Methyl-thiol-1-propanol	0.101 ± 0.006^{a}	0.181 ± 0.010^{b}	0.256 ± 0.005^{c}	0.902 ± 0.042^{d}	0.390 ± 0.017^{e}
% Thiols	0.00	0.00	0.00	0.00	0.00
**Phenols**					
Guaiacol	0.364 ± 0.002^{a}	0.588 ± 0.023^{b}	0.429 ± 0.002^{c}	0.362 ± 0.011^{a}	0.393 ± 0.025^{a,c}
Acetovanillone	0.653 ± 0.007^{a}	0.080 ± 0.001^{b}	0.143 ± 0.006^{c}	0.453 ± 0.048^{d}	0.355 ± 0.015^{e}
4-Hydroxybenzeneethanol	3.415 ± 0.087^{a}	4.622 ± 0.173^{b}	4.511 ± 0.070^{b}	4.971 ± 0.048^{c}	3.838 ± 0.024^{d}
4-vinylguayacol	0.852 ± 0.116^{a}	1.255 ± 0.092^{b}	0.907 ± 0.093^{a}	1.244 ± 0.034^{b}	2.137 ± 0.089^{c}
2,6-Dimethoxyphenol	0.138 ± 0.008^{a}	0.888 ± 0.088^{b}	0.178 ± 0.007^{a}	1.128 ± 0.020^{c}	0.618 ± 0.026^{d}
Total	5.421 ± 0.221	7.433 ± 0.377	6.168 ± 0.177	8.159 ± 0.161	7.340 ± 0.179
% Phenols	0.01	0.02	0.01	0.01	0.01
**Terpenes**					
Linalool	0.247 ± 0.019^{a}	0.231 ± 0.020^{a}	0.501 ± 0.016^{b}	0.329 ± 0.015^{c}	1.455 ± 0.117^{d}
% Terpenes	0.00	0.00	0.01	0.00	0.01
**Lactones**					
2,3-Dihydro-Benzofuran	0.450 ± 0.004^{a}	0.669 ± 0.040^{b}	0.870 ± 0.045^{c}	0.811 ± 0.097^{c}	0.872 ± 0.011^{c}
% Lactones	0.00	0.00	0.00	0.00	0.00

SD48h: sun-dried grapes during 48h. SD96h: sun-dried grapes during 96h. CH48h: climatic chamber drying during 48h. CH96h: climatic chamber drying during 96h. Different letters in superscript mean significant differences ($p < 0.05$) between samples determined by a two-way ANOVA according to Bonferroni's multiple range (BSD) test.
